# Normal Values for Left Ventricular Mass in Relation to Lean Body Mass in Child and Adolescent Athletes

**DOI:** 10.1007/s00246-018-1982-9

**Published:** 2018-09-12

**Authors:** Hubert Krysztofiak, Marcel Młyńczak, Andrzej Folga, Wojciech Braksator, Łukasz A. Małek

**Affiliations:** 1 Mossakowski Medical Research Centre, Polish Academy of Sciences, 5 Pawinskiego Street, 02-106 Warsaw, Poland; 2 National Centre for Sports Medicine, 78 Pory Str, 02-757 Warsaw, Poland; 3 Institute of Metrology and Biomedical Engineering, Faculty of Mechatronics, Warsaw University of Technology, 8 Andrzeja Boboli Str, 02-525 Warsaw, Poland; 4 Department of Sports Cardiology and Noninvasive Cardiovascular Imaging, Second Faculty of Medicine, Medical University of Warsaw, 8 Kondratowicza Str, 03-258 Warsaw, Poland; 5 Faculty of Rehabilitation, Józef Piłsudski University of Physical Education, 34 Marymoncka Str, 00-968 Warsaw, Poland

**Keywords:** Left ventricular mass, Lean body mass, Echocardiography, Children, Sport, Normal values

## Abstract

It has been demonstrated that regular sport activity in children leads to physiological changes in the heart including increased left ventricular (LV) myocardial thickness and mass (LVM). The aim of the study was to establish the first specific normal values of LVM for child and adolescent athletes. Parasternal long-axis, 2D-guided echocardiographic measurements were obtained from a group of 791 Caucasian child athletes (age 5–18 years, 58.7% boys). For the preparation of normative data, LVM-for-lean body mass (LBM) reference curves were constructed using the LMS method. Then, a simple correlation plot was constructed to analyse the concordant and discordant indications of left ventricular hypertrophy (LVH), defined as LVM-for-LBM above the 95th percentile, according to the newly created and previously published normative data on LVM-for-LBM in the general population of children. Reference scatter plots of LVM-for-LBM for boys and girls in the analysed group of children practicing sports were presented, showing mean values of LVM and z-scores. The application to the studied group of reference centiles established for the general population of children would lead to false positive misclassification of increased LVH in 5.8% of the girls and 17.0% of the boys. We present the first specific normative data for LV mass in relation to lean body mass in Caucasian children and adolescents engaged in regular sport activities. The application of specific normative data for LV mass results in fewer false positive findings of left ventricular hypertrophy in this group than that of reference values for general paediatric population.

## Introduction

It has been demonstrated that regular athletic activity in children leads to physiological changes in the heart similar to adult athlete’s heart, but usually less prominent [1–5]. Those changes mainly include increased left ventricular (LV) myocardial thickness and LV mass (LVM) [5–7]. We have recently constructed normative data for basic echocardiographic linear dimensions of the heart in a large group of child and adolescent athletes (own unpublished data) and compared them to the previously published normative data for the paediatric population [8]. Z-scores differed significantly between these two populations for all the analysed parameters. Two of the most evident differences were found for interventricular septal thickness and posterior wall thickness. As these parameters together with LV cavity dimension influence LV mass, it seems obvious that LV mass would be larger in child athletes than in the general paediatric population [9]. However, there is no specific normative data for LV mass in this subset of children. Most recent reference centiles for LV mass relative to lean body mass (LBM) in the general paediatric population were published a few years ago by Foster et al. [10]. The authors found that LBM is the strongest determinant of LV mass, better than age, height, weight or body surface area (BSA) [11, 12]. They also postulated that there is a linear relationship between LV mass and LBM, which might explain the increase of LV mass in relation to increased LBM due to training. It is also hypothesized, that in obese children LV mass may rise as LBM increases due to “training” caused by carrying extra fat [10].

Therefore, the aim of the study was to establish specific normal values of LV mass for child and adolescent athletes, as well as to challenge the hypothesis of a linear relationship between LV mass and LBM in relation to physical training.

## Methods

### Study Group

This retrospective study assessed healthy, non-obese Caucasian children and adolescents (327 girls and 464 boys, ages 5–18 years). Non-obesity was defined as a body mass index (BMI) below the 85th percentile according to recommendations and as used in previous studies on normal LV mass values in children [10, 12, 13].

All of the studied children were engaged in regular athletic training at the local or national level (mainly soccer, track and field, basketball, swimming and martial arts). Detailed characteristics of the study group, including age, height, weight, BSA, body mass index (BMI), LBM, LV mass, LV cavity dimension, posterior wall thickness, interventricular septal thickness and training volume, are presented in Table 1. Body surface area was calculated according to the Haycock formula [14].

**Table 1:** Study group characteristics

Parameter	Male subjects	Female subjects
*N* (%)	464 (58.7%)	327 (41.3%)
Age (years)	12 (6)	12 (5)
Height (m)	1.59 (0.35)	1.53 (0.22)
Weight (kg)	46.25 (30.40)	41.80 (20.35)
BSA (m^2^)	1.43 (0.61)	1.33 (0.40)
BMI (kg/m^2^)	18.54 (4.76)	17.88 (4.23)
LBM (kg)	34.85 (23.39)	29.85 (13.26)
LVM (g)	113.88 (70.69)	93.46 (39.05)
LV cavity dimension (mm)	45 (8)	42 (6)
Interventricular septal thickness (mm)	8 (2)	8 (1)
Posterior wall thickness (mm)	8 (2)	7 (1)
Training volume (min)*	270 (270)	240 (180)

All unit-bearing values are represented as “median (interquartile range)”

*BMI* body mass index, *BSA* body surface area, *LBM* lean body mass, *LV* left ventricular, *LVM* left ventricular mass

*The training volume determines the level of involvement in a sport and was estimated as the product of the average number of training sessions per week and the average duration of a single training session

### Echocardiography

All study participants had undergone transthoracic echocardiography as part of periodic pre-participation evaluation (PPE) due to innocent heart murmurs or suspicion of abnormal electrocardiographic findings. The studies were performed at the National Centre for Sports Medicine between 2013 and 2017. Children thus found to have significant acquired or congenital heart diseases affecting normal heart size and haemodynamics were excluded. Echocardiograms were performed by two experienced sonographers using a commercially available ultrasound scanner (Toshiba Aplio 400, Toshiba Medical Systems Europe, Zoetermeer, the Netherlands), according to recent guidelines [15]. All measurements were taken in 2-dimensional parasternal long-axis view (PLAX) in end-diastole. The measurements were taken from inner edge to inner edge and reported to within 1 mm. Persons exhibiting ambiguous results of any measurement were excluded from the initial study group. LV mass (in grams) was calculated using the Devereux equation: LVM = 0.8{1.04[(LV cavity dimension + posterior wall thickness + interventricular septal thickness)^3^ − (LV cavity dimension)^3^]} + 0.6 [9].

### Ethical Considerations

The study procedure was approved by the Ethics Committee of the Medical University of Warsaw (AKBE/75/17). Parents or legal guardians of each subject had signed the consent form for the PPE, including a statement agreeing to the use of the results for scientific purposes.

### Statistical Methods

For the calculation of LBM, previously published equations were used [16]. For male subjects, ln(LBM) = − 2.8990 + 0.8064 × ln(height) + 0.5674 × ln(weight) + 0.0000185 weight^2^ − 0.0153 (BMI z-score)^2^ + 0.0132 × age. For female subjects: ln(LBM) = − 3.8345 + 0.954 × ln(height) + 0.6515 × ln(weight) − 0.0102 (BMI z-score)^2^.

For the preparation of normative data, we constructed LVM-for-LBM reference curves using the LMS method as described previously [17, 18]. The curves present mean values in the studied population as well as z-scores.

In order to analyse the concordant and discordant indications of left ventricular hypertrophy (LVH), defined as LVM-for-LBM above the 95th percentile (z-score + 1.64), according to the newly created and previously published normative data [10], we constructed a simple correlation plot divided into quadrants based on the z-score cut-off value that defines LVH [10]. We estimated the parameters describing the percentages of all results found in the concordant and discordant areas.

All calculations used R software (version 3.4.2, “Short Summer”; R Foundation, Vienna, Austria, http://www.r-project.org), along with external packages. For all statistical tests, a significance level of *α* = 0.05 was applied.

## Results

### Normative Data on LVM-for-LBM in Children Practicing Sports

**Fig. 1:**
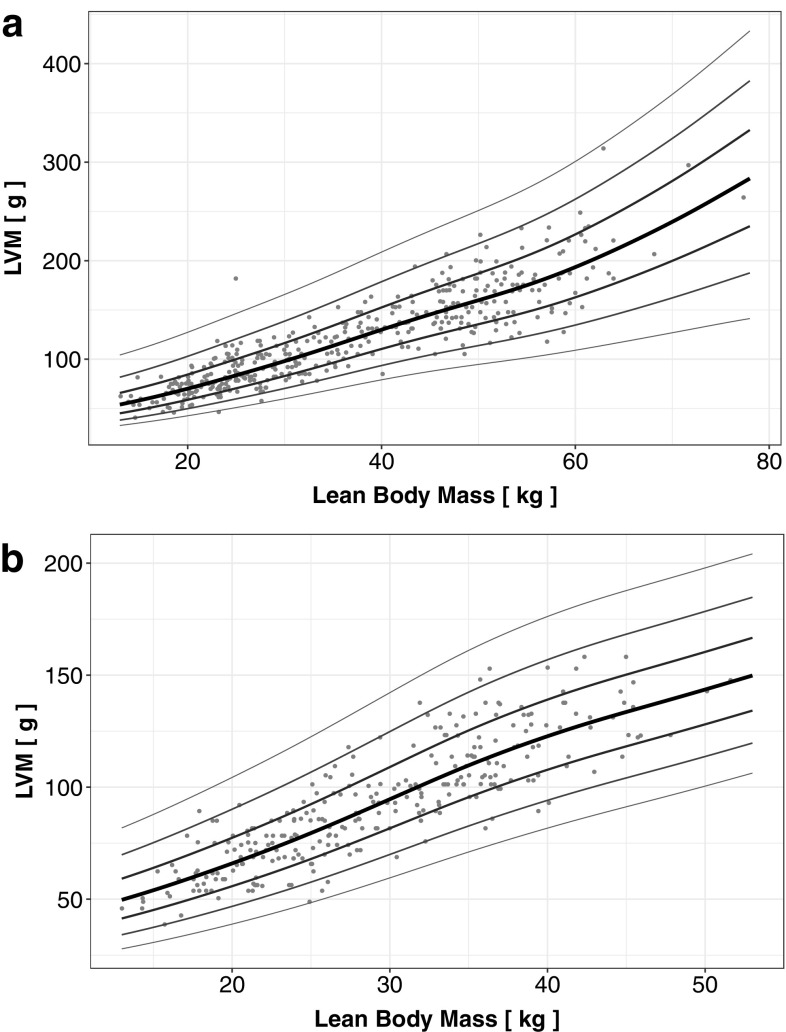
Z-scores of LVM-for-LBM in children practicing sports: **a** boys, **b** girls

Separate reference scatter plots of LVM-for-LBM for boys and girls in the analysed group of children practicing sports are presented in Fig. 1a—boys, b—girls. The black line represents the estimated mean z-score = 0, while other lines represent z-score values of ± 1, 2 and 3 (standard deviations from the mean).

### Comparison of LVH Indications by the LVM-for-LBM Normative Data for the General Population and in Child Athletes

**Fig. 2:**
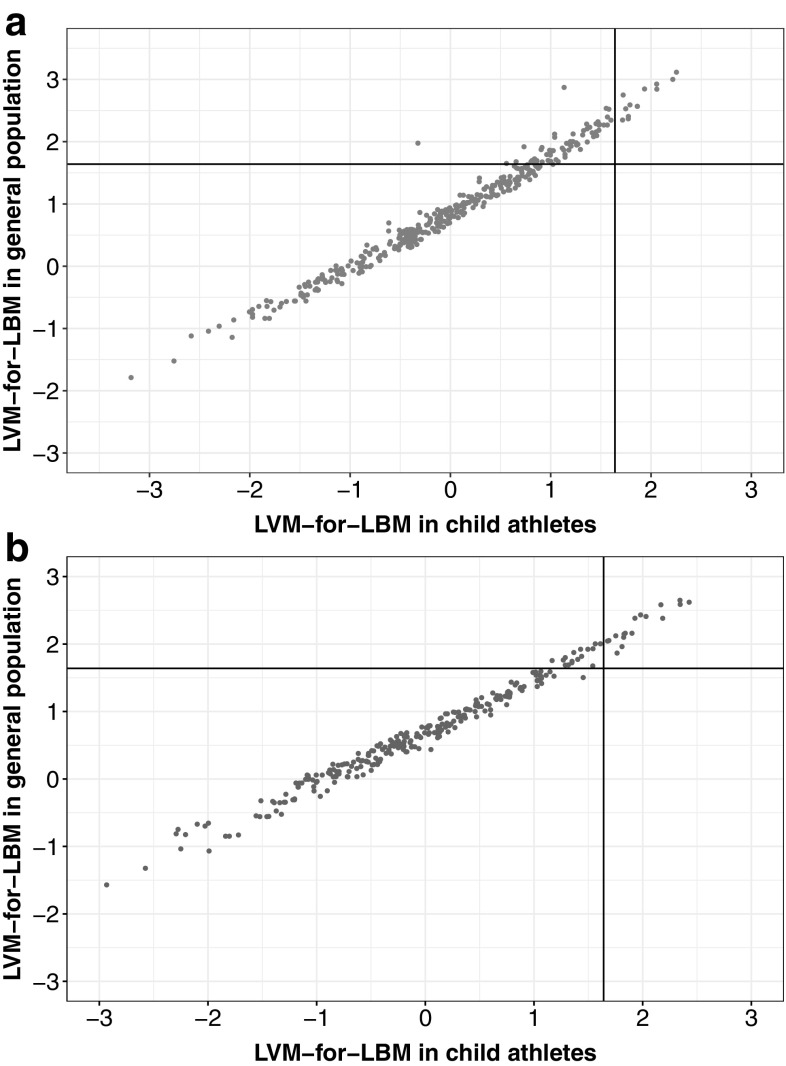
Simple correlation plots divided into quadrants based on the z-score cut-off value that defines LVH (z-score + 1.64) showing concordant and discordant indications of LVH according to z-scores computed on normative data obtained for child athletes (LVM-for-LBM in child athletes) and for the general population of children (LVM-for-LBM in general population) [10]: **a** boys, **b** girls

A simple correlation plot divided into quadrants based on the z-score cut-off value that defines LVH (z-score + 1.64) to detect the percentage of concordant and discordant indications of LVH according to z-scores computed based on the Foster et al. group (general paediatric population) and on our group (child athletes) is shown in Fig. 2. Percentage agreement in LVH indications between both groups is presented in Table 2. This shows that the use of reference centiles from the general population would lead to false positive detection of increased LV mass in 5.8% of girls and 17.0% of boys. LVH was found in 6.0% of girls and 3.8% of boys when the newly proposed reference values were applied.

**Table 2:** Agreement between identifications of LVH using LVM-for-LBM normative data for the general population and in child athletes

		Female		Male	
		LVM-for-LBM in general population			
		No LVH (%)	LVH (%)	No LVH (%)	LVH (%)
LVM-for-LBM in child athletes	No LVH	88.1	5.8	79.2	17.0
	LVH	0.0	6.1	0.0	3.8

*LBM* lean body mass, *LVH* left ventricular hypertrophy, *LVM* left ventricular mass, *LVM-for-LBM* LVM realtive to LBM

## Discussion

Previous echocardiographic studies demonstrated that even a relatively short 10-week physical training programme leads to increased LV wall thickness in children [7]. Longer physical training in children causes other adaptive changes, such as right ventricular or atrial enlargement, which have been summarized in detail elsewhere [2, 4]. Also, we had recently confirmed the increases in LV wall thickness and LV mass related to prolonged physical training in pre-adolescent soccer players with cardiac magnetic resonance, which is considered a gold standard for the assessment of LV volume, systolic function and mass [5]. Therefore, we decided to obtain specific normative data on LV mass for child athletes, which has never been done before. For that reason, we used methodology similar to Foster et al. who published the most recent normative data on LV mass in the general paediatric population [10]. Those data were based on a group of subjects ages 5–18 years and included scaling of LV mass to LBM as the best indexing method, limitation of the dataset to non-obese children, use of the LMS method for construction of the centile curves, as well as presentation of separate data for boys and girls. The only deviation from these features was the presentation of data in the form of z-scores instead of centile curves. The choice of such methodology allowed comparison of our normative data with those of the general population.

We have checked how the new reference values change the indication of LVH. As expected, the use of specific normal values for junior athletes leads to avoidance of false positive LVH indications in as many as 5% of girls and 17% of boys.

According to the Fosters et al. data, indication of LVH was almost twice as frequent in males (20.8%) as in females (11.9%) [10]. Application of our specific normative data reversed that relation by excluding cases of physiological adaptation and demonstrated that LVH (defined as LV mass above the 95th percentile) is more frequent in females (6.1%) than in males (3.8%). This may be surprising, as some previous research indicated that indexed LV mass is greater in male early-adolescent, non-professional athletes [19]. Also, the prevalence of LVH had been shown to be 2.6 times greater in male than in female athletes [4]. These differences may be due to at least two facts. (1) other studies used a different method of LV mass indexation (BSA vs. LBM), (2) most studies indicating higher prevalence of LVH in boys than in girls used a unified definition of LVH (LV wall thickness > 12 mm, instead of LV mass > 95th percentile for a specific sex) [4, 6]. Finally, differing pubertal ages of the studied children could also play a role, as markedly greater LVM growth in males usually starts to appear close to pubertal development [4, 19].

Our data question the hypothesis that there is a continuous, linear relation between LV mass and LBM in normal-weight children, as was presented by Foster et al. [10]. We have demonstrated that LV mass increases more than LBM in relation to regular physical activity, and this may lead to false positive findings of LVH. However, it remains to be elucidated whether the linear relation between LV mass and LBM is preserved in plain strength sports, where larger LBM changes are expected. The majority of sport disciplines practiced by children, as in our case, consist of mixed sports where, it seems, the LVM-LBM relation is not linear.

Our study has some limitations. Firstly, we used 2-dimensional echocardiographic measurements, which are currently the supported method of baseline measurements, while the compared study used motion (M) mode views [15]. As LV mass is calculated from those measurements, this could have influenced the results. Secondly, the study subjects were all Caucasian children and adolescents, while there has been no information regarding subjects’ ethnicity in Fosters et al. study. Ethnic variability may influence the LVM normative data, so our reference values should be applied to Caucasian population [4]. Finally, we studied a mixed group of young athletes, engaged in different sports, which may influence the LV mass increase in a different way. Nevertheless, majority of training in the young athletes we studied was focused on general development with building of aerobic base and physical activity skills. Therefore, the type, volume and intensity are comparable and general effect on the heart is similar.

## Conclusions

We present the first specific normative data for LV mass in relation to lean body mass in Caucasian children and adolescents engaged in regular athletics (at the local or national levels). The application of specific normative data for LV mass results in fewer false positive findings of left ventricular hypertrophy in this group than that of reference values for the general paediatric population.
